# Lactation and cardiovascular risk factors in mothers in a population-based study: the HUNT-study

**DOI:** 10.1186/1746-4358-7-8

**Published:** 2012-06-19

**Authors:** Siv T Natland, Tom I L Nilsen, Kristian Midthjell, Lene F Andersen, Siri Forsmo

**Affiliations:** 1 Department of Public Health and General Practice, Norwegian University of Science and Technology, PO Box 8904 MTFS, 7491, Trondheim, Norway; 2 Department of Human Movement Science, Norwegian University of Science and Technology, Dragvoll, NO-7491, Trondheim, Norway; 3 HUNT Research Centre, Norwegian University of Science and Technology, Forskningsveien 2, N-7600, Levanger, Norway; 4 Institute of Basic Medical Sciences, Postboks 1046, Blindern, 0317, Oslo, Norway

## Abstract

**Background:**

Lactation has beneficial short term effects on maternal metabolic health, but the long term effects are less well known.

**Methods:**

We studied the association between lifetime duration of lactation and cardiovascular risk factors in mothers later in life among 21,368 parous women aged 20 to 85 years attending the second Nord-Trøndelag Health Study (HUNT2) in 1995–1997, Norway, a cross-sectional population-based study. General linear modelling was used to calculate mean values of known cardiovascular risk factor levels in five categories of lifetime duration of lactation. Logistic regression was conducted to estimate odds ratios of hypertension, obesity and diabetes.

**Results:**

Among women aged 50 years or younger, lifetime duration of lactation was significantly and inversely associated with body mass index (*P*-trend, < 0.001), waist circumference (*P*-trend, < 0.001), systolic and diastolic blood pressure (both *P*-trends, < 0.001), and serum levels of triglycerides, total cholesterol and low density lipoprotein cholesterol (all *P*-trends, < 0.001) after adjustment for covariates. Parous women aged 50 years or younger who had never lactated had higher prevalence of hypertension, obesity and diabetes. In this age group, compared to women who had lactated for 24 months or more, parous women who had never lactated had an OR for hypertension of 1.88 (95% CI 1.41, 2.51), an OR for obesity of 3.37 (95% CI 2.51, 4.51) and an OR for diabetes of 5.87 (95% CI 2.25, 15.3). Among women older than 50 years there were no clear associations.

**Conclusion:**

Lifetime duration of lactation was associated with long term reduced cardiovascular risk levels in mothers aged 50 years or younger.

## Background

Cardiovascular disease is the most common cause of death in women in the western world, and among its major modifiable risk factors are hypertension, dyslipidaemia, obesity and type 2-diabetes [1]. Lactation is a factor unique to women that may be associated with all these risk factors, and several studies have shown that it may affect them favourably [2-4]. Moreover, such advantages may persist several years post-weaning [5-13]. However, previous studies evaluating the association between lactation and maternal cardiovascular health have suffered from being short-term, having small sample sizes, or samples from selected populations or populations with low breastfeeding rates. Norway has one of the highest breastfeeding rates in Europe, with 80% of the infants still being breastfed at six months [14]. We have therefore studied the association between lifetime duration of lactation and maternal cardiovascular risk factors later in life in a large unselected Norwegian population sample (about 35,000 women) in which breastfeeding was common and breastfeeding duration was long.

## Methods

### Study population

The Nord-Trøndelag Health Study (HUNT) is a population-based health survey aiming at the total adult population >19 years of age in the county of Nord-Trøndelag, Norway. Data collection and methods have been described in detail elsewhere [15]. Briefly, the second HUNT study (HUNT2) took place between 1995 and 1997, and included two self-administered questionnaires and a clinical examination including standardised measurements of height, weight, waist circumference and blood pressure, as well as non-fasting measurements of blood glucose and serum lipids. The first questionnaire was sent by mail along with an invitation for a clinical examination. The questionnaire form included questions about general health and lifestyle, and the participants were requested to bring it to the physical examination. A second, more detailed questionnaire containing queries on number of live births and corresponding lactation history, as well as illnesses, medical treatment, lifestyle and socio-economic factors was distributed during the examination, to be completed at home and returned by mail.

Among 47,312 women invited to HUNT2, a total of 35,280 (75.5%) women participated. For this study, reasons for exclusions were non-response to the second questionnaire (n = 5,061), current pregnancy (n = 605), age > 85 years (n = 343), non-attendance at the clinical examination (n = 355), self-report of infarction (n = 1), stroke (n = 9), angina (n = 12), diabetes prior to the first live birth (n = 43), less than one year since last child birth (n = 257) or unknown lactation history (n = 2 206), leaving 26,388 women as eligible for the analyses, of whom 21,368 women had given birth to at least one child. For the analyses of blood pressure data we further excluded those who reported current or previous antihypertensive medication (n = 3 061 among parous women, n = 682 among nulliparous women). For the analyses of low density lipoprotein cholesterol we excluded those with triglyceride concentrations of 4.5 mmol/L or more (n = 321 among parous women, n = 78 among nulliparous women).

### Lactation history

Lactation history was self-reported by the women in the second questionnaire. For each live birth, the women reported the year of birth and corresponding lactation duration in whole months (*“How many months did you breastfeed?”*). Lifetime duration of lactation was calculated as the sum of lactation duration for all live births and categorised into five levels (none, 1–6, 7–12, 13–23, and ≥24 months).

### Clinical measurements

Height was measured without shoes to the nearest 1.0 cm and weight wearing light clothing to the nearest 0.5 kg. Body mass index was calculated as weight (kg) divided by the squared value of height (m^2^). Waist circumferences were measured with a flexible steel band with the participants standing upright, and the numbers were rounded to the nearest 1.0 cm. Waist circumference was measured horizontally at the height of the umbilicus [15].

Blood pressure was measured by specially trained nurses or technicians with oscillometric Dinamap 845 XT (® Critikon, Tampa, FL) after adjustment of the cuff size according to the arm circumference. After an initial two minutes’ rest, the blood pressure was automatically measured three times at intervals of one minute. In this study, we used the mean value of the second and third measurement of systolic and diastolic blood pressure.

The blood sample (non-fasting) drawn from all participants was centrifuged at the screening station and, on the same day, transported in a cooler to the laboratory. Serum lipids were analysed at the Central Laboratory, Levanger Hospital, Nord-Trøndelag Hospital Trust, using a Hitachi 911 Autoanalyser (Hitachi, Mito, Japan), applying reagents from Boehringer Mannheim (Mannheim, Germany). Total serum cholesterol, high density lipoprotein (HDL) cholesterol and triglycerides were measured by an enzymatic colorimetric method, and HDL cholesterol was measured after precipitation with phosphotungstate and magnesium ions. Glucose was measured using an enzymatic hexokinase method. The day-to-day coefficients of variation were 1.3-1.9% for total cholesterol, 2.4% for HDL-cholesterol, 0.7-1.3% for triglycerides and 1.3-2.0% for glucose.

Low density lipoprotein (LDL) cholesterol was calculated using the Friedewald formula: LDL cholesterol = total serum cholesterol – HDL cholesterol – one-fifth of the triglyceride concentration [16]. LDL was only calculated in participants with triglyceride concentrations lower than 4.5 mmol/L.

### Analyses

In order to examine whether the effects of lactation duration were modified by age, we conducted analysis stratified by age (≤ 50 years and > 50 years) and also tested for statistical interaction by including a product term of lactation duration and age ±50 years in the regression model. We used a general linear model to calculate mean values of body mass index, waist circumference, systolic and diastolic blood pressure, serum lipid levels and blood glucose levels in five categories of lactation, and to estimate adjusted mean difference with 95% confidence intervals (CI) between the categories. Previous studies have shown beneficial maternal metabolic effects with increasing duration of lactation [6,11,17]. Hence, lifetime duration of lactation for ≥24 months, the category with the longest lactation duration, was used as a reference category, as this was assumed to be the most beneficial lactation duration. Lipid and glucose concentrations were log-transformed due to non-normal distribution, and hence we calculated geometric means and crude and adjusted differences in percent between the categories for each lipid and for glucose. *P*-values for linear trend were calculated *first* across the five categories of lactation duration and then across four categories of lactation duration, excluding the ‘never lactated’ group, by treating the categories as an ordinal variable in the regression model. All associations were adjusted for potential confounding, with maternal age, education (primary school, secondary school, college/university and unknown), smoking status (current, former or never smoked), hours of physical activity per week (no activity, <3 hours light or <1 hour hard activity, >3 hours light or 1 hour hard activity, >1 hour hard activity, and unknown), marital status (unmarried, divorced, widowed and married/cohabiting) and parity (1, 2, 3 or ≥ 4 children). In analyses of serum lipids and blood glucose we also adjusted for time since last meal. In supplementary analyses, we adjusted first for time since last birth, and then for body mass index. We also did the analyses described above comparing nulliparous and parous women.

In additional analyses, we used logistic regression to estimate crude and adjusted odds ratios (ORs) with 95% CIs of hypertension (≥ 140/90 mmHg or current antihypertensive treatment), obesity (body mass index ≥ 30 kg/m^2^), and diabetes (‘yes’ versus ‘no’ to the question ‘Do you have or have you had diabetes?’ or blood glucose ≥ 11.1 mmol/L) associated with five categories of lactation duration. We also did corresponding analyses comparing nulliparous and parous women.

All statistical tests were two-sided, and all analyses were performed using SPSS for Windows (version 16, SPSS Inc., Chicago; IL, USA).

### Ethical approval

The study was approved by the Norwegian Regional Committees for Medical and Health Research Ethics and by the Norwegian Data Inspectorate. Informed consent was given by all participants in the HUNT-study.

## Results

The parous women had a mean age of 50.4 years when attending HUNT2, they reported a median parity of two live births, a median lifetime duration of lactation of 13 months and time since the last delivery was on average 21.1 years (data not shown). The majority (96.7%) of the 21,368 parous women had breastfed one or more children, and approximately one in five women (21.4%) reported a lifetime duration of lactation longer than 24 months. Across the lactation categories, there were significant differences in maternal age, educational level, smoking status, the level of physical activity, marital status and parity in our study (Table 1). Furthermore, the variables that we a priori considered as confounders, namely: educational level, smoking status, level of physical activity, marital status and parity, were all associated with the outcome variables in our study (data not shown).

**Table 1:** Characteristics of women in the HUNT2-study, Norway, 1995–97 (N = 26,388)

	**Nulliparous women**	**Parous women**				
		**Lifetime duration of lactation (months)**				
**Variables**^**a**^	**n = 5,020**	**0 n = 705**	**1 – 6 n = 4,421**	**7 – 12 n = 5,401**	**13 – 23 n = 6,266**	**≥ 24 n = 4,575**
Age, yrs, mean (SD)	44.7 (21.6)	50.7 (13.7)	48.4 (14.5)	49.3 (14.9)	50.1 (14.8)	53.8 (15.7)
Age at delivery of first child, yrs, mean (SD)	-	24.2 (5.1)	23.3 (4.4)	23.3 (4.2)	23.4 (3.9)	23.2 (3.7)
Parity,						
Para 1 (%)	-	29.9	30.8	17.2	2.5	0.4
Para 2 (%)	-	38.3	45.1	44.0	45.1	12.9
Para 3 (%)	-	21.7	17.4	26.7	35.0	41.2
Para 4 or greater (%)	-	10.1	6.8	12.0	17.5	45.5
University/college education (%)^b^	26.4	13.2	13.4	18.0	21.7	22.4
Never smoked (%)	62.2	37.3	36.7	45.7	50.9	60.5
High physical activity^c,d^ (%)	28.7	14.6	17.6	18.2	20.9	18.2
Unmarried/divorced (%)	61.6	24.4	28.7	23.2	16.5	11.1
Hypertension^e^ (%)	37.5	47.2	36.3	37.5	37.5	44.9
Obesity^f^ (%)	17.0	27.9	18.7	18.0	16.3	21.2
Diabetes (%)^g^	3.0	4.4	1.9	2.0	2.4	3.7

Abbreviation: SD, standard deviation.

^a^Continuous variables presented as mean (SD), categorical variables presented as % in each category of lactation.

^b^ Number of women in the category unknown: n = 823.

^c^ High physical activity defined as ≥ 1 hour hard physical activity per week.

^d^ Number of women in the category unknown: n = 2,059.

^e^Hypertension defined as ≥ 140/90 mmHg or current antihypertensive medication.

^f^ Obesity defined as body mass index ≥ 30 kg/m^2^.

^g^Diabetes defined as blood glucose ≥ 11.1 mmol/L or self reported diabetes in the questionnaire.

There was evidence of statistical interaction between age at participation (± 50 years) and lactation duration for several of the outcome variables under study (*P*-values from interaction tests; < 0.001 for BMI; < 0.001 for waist circumference; 0.043 for systolic blood pressure; < 0.001 for triglycerides; < 0.001 for total cholesterol, and 0.011 for HDL-cholesterol). Thus, the remainder of the analyses were stratified by age. Overall, there was an inverse association between lifetime duration of lactation and both body mass index (*P*-trend, < 0.001) and waist circumference (*P*-trend = 0.01) among women 50 years of age or younger (Table 2). After adjusting for potential confounders, women 50 years of age or younger who reported no lactation had a body mass index that was 2.5 kg/m^2^ (95% CI 2.0, 3.0) higher and a waist circumference that was 5.3 cm (95% CI 4.2, 6.5) wider than the reference group of women who had lactated ≥ 24 months (Table 2). Adjusting for time since last birth did not change these estimates. Among women older than 50 years, there were no significant relations between duration of lactation and body mass index. However, women older than 50 years who reported no lactation had a waist circumference that was 1.5 cm wider than the reference group who had lactated ≥ 24 months.

**Table 2:** Body mass index and waist circumference in nulliparous and parous women (n = 26,388)

	***Women ≤ 50 years of age (n = 14,677)***				***Women > 50 years of age (n = 11,711)***			
	**No.**	**Crude mean**	**Adjusted mean**^**a**^	**95 % CI**	**No.**	**Crude mean**	**Adjusted mean**^**a**^	**95 % CI**
**BMI (kg/m**^**2**^**)**								
Nulliparous women	3,011	24.8	25.8	25.5, 26.1	2,009	27.3	26.9	26.6, 27.1
Parous women	11,666	25.4	25.6	25.3, 25.9	9,702	27.5	27.3	27.1, 27.5
*P-*value^b^			0.026				<0.001	
*Months of lifetime lactation*								
Never	360	27.5	27.6	27.1, 28.1	345	27.7	27.7	27.2, 28.3
1–6	2,587	25.7	26.1	25.8, 26.5	1,834	27.1	27.2	26.9, 27.6
7–12	3,038	25.4	25.7	25.4, 26.0	2,363	27.2	27.2	26.9, 27.5
13–23	3,513	25.1	25.3	25.0, 25.6	2,753	27.4	27.3	27.0, 27.6
≥ 24	2,168	25.1	25.1	24.8, 25.5	2,407	28.2	27.6	27.2, 27.9
*P*- trend^c^			<0.001				0.542	
*P*- trend^d^			<0.001				0.675	
**Waist cirmcumference (cm)**								
Nulliparous women	3,012	76.1	79.2	78.5, 80.0	2,009	85.3	84.2	83.6, 84.7
Parous women	11,919	78.8	79.3	78.6, 80.0	9,702	85.0	85.0	84.5, 85.5
*P-*value^b^			0.723				0.004	
*Months of lifetime lactation*								
Never	360	83.4	83.9	82.6, 85.2	345	86.3	86.9	85.7, 88.2
1–6	2,587	79.5	80.5	79.7, 81.4	1,834	84.3	85.1	84.3, 85.9
7–12	3,038	78.8	79.7	78.9, 80.5	2,363	84.3	84.7	83.9, 85.5
13–23	3,513	78.1	78.9	78.1, 79.8	2,753	84.4	84.6	83.8, 85.4
≥ 23	2,168	78.4	78.6	77.7, 79.4	2,407	86.8	85.4	84.5, 86.2
*P*- trend^c^			<0.001				0.03	
*P*- trend^d^			<0.001				0.085	

Abbreviations: No, number; CI, confidence interval; BMI, body mass index.

^a^ For nulliparous women: Adjusted for maternal age, smoking status, physical activity, education and marital status. For parous women: Adjusted for maternal age, smoking status, physical activity, education, marital status and parity.

^b^*P*-value between nulliparous vs parous women. Adjusted for maternal age, smoking status, physical activity, education and marital status.

^c^*P*-trend across all five categories of lifetime lactation duration, including the category “never”.

^d^*P*-trend across four categories of lifetime lactation duration, excluding the category “never”.

**Figure 1:**
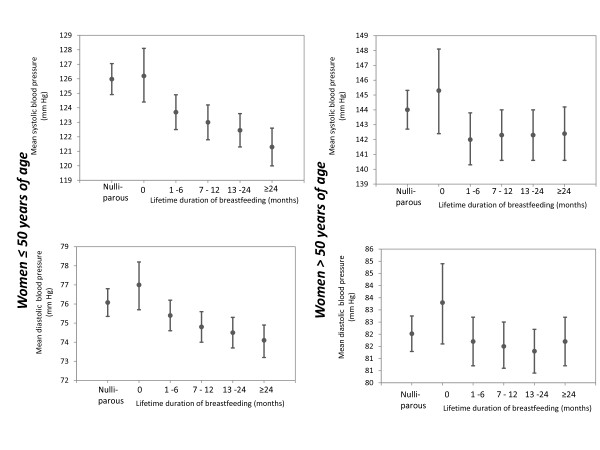
**Adjusted mean systolic and diastolic blood pressure (with 95%CI) in nulliparous and parous women.** For nulliparous women (n = 4,338): Adjusted for age, smoking, physical activity, education and marital status. For parous women (n =18,307): Adjusted for age, smoking, physical activity, education, marital status and parity. (Number of women in each category of lifetime duration of breastfeeding among women ≤ 50 years of age n = 11,150): 0 months: n = 335, 1–6 months: n = 2,455, 7–12 months: n = 2,893, 13–23 months: n = 3,380, 24+ months: n = 2,087. Number of women in each category of lifetime duration of breastfeeding among women > 50 years of age (n = 7,157): 0 months: n = 251, 1–6 months: n = 1,429, 7–12 months: n = 1,770, 13–23 months: n = 2,057, 24+ months: n = 1,650).

A similar pattern was observed in age-stratified analysis of systolic and diastolic blood pressure, shown in Figure 1. In multi-adjusted analysis, women 50 years of age or younger who had never lactated had 4.9 mmHg (95% CI 3.2, 6.6) higher systolic blood pressure and 2.9 mmHg (95% CI 1.8, 4.1*)* higher diastolic blood pressure than women who had lactated ≥ 24 months (both *P*-trends, < 0.001). Among women older than 50 years, there were no significant relationships between duration of lactation and systolic or diastolic blood pressure. Additional adjustment for body mass index and time since last birth attenuated the estimates of both systolic and diastolic blood pressure among women 50 years or younger, whereas the estimates among women older than 50 years remained largely similar (data not shown).

The analysis of lifetime duration of lactation and levels of triglycerides, total cholesterol and LDL-cholesterol also showed an inverse pattern and an apparent dose–response relationship (all *P*-trends, < 0.001) among women 50 years or younger, whereas among women older than 50 years no significant associations were found. In analyses of log-transformed lipid values, women 50 years or younger who had never lactated had 17% (95% CI 11, 24) higher triglyceride levels, 5% (95% CI 3, 7) higher total cholesterol levels and 8% (95% CI 4, 11) higher LDL-cholesterol levels than women who had lactated ≥ 24 months (Table 3). Additional adjustments for time since last birth did not change these estimates, whereas additional adjustments for body mass index attenuated the estimates (data not shown). The results for HDL cholesterol showed a somewhat different pattern. Women 50 years or younger who had never lactated had 4% (95% CI 1, 6) lower HDL cholesterol levels than women who had lactated ≥ 24 months (*P*-trend, 0.008). Additional adjustments for time since last birth did not change these estimates, whereas no associations remained after adjustments for body mass index. As in the analyses of the other serum lipids, there were no significant associations among women older than 50 years.

**Table 3:** Triglycerides, total-, HDL- and LDL-cholesterol and blood glucose (log-transformed) in nulliparous and parous women (n = 26,388)

	***Women ≤ 50 years of age (n = 14,677)***				***Women > 50 years of age (n = 11,711)***			
	**No.**	**Crude geometric mean**	**Adjusted geometric mean**^**a**^	**95 % CI**	**No.**	**Crude geometric mean**	**Adjusted geometric mean**^**a**^	**95 % CI**
**Triglycerides (mmol/L)**								
Nulliparous women	3,011	1.09	1.21	1.17, 1.26	2,009	1.66	1.58	1.54, 1.62
Parous women	11,666	1.14	1.16	1.12, 1.20	9,702	1.63	1.63	1.59, 1.66
*P-*value^b^			<0.001				0.02	
*Months of lifetime lactation*								
Never	360	1.34	1.30	1.22, 1.38	345	1.64	1.70	1.61, 1.80
1–6	2,587	1.22	1.22	1.17, 1.27	1,834	1.55	1.61	1.55, 1.67
7–12	3,038	1.15	1.17	1.13, 1.22	2,363	1.59	1.63	1.57, 1.68
13–23	3,513	1.11	1.14	1.10, 1.19	2,753	1.64	1.65	1.59, 1.70
≥ 24	2,168	1.08	1.11	1.07, 1.16	2,407	1.70	1.63	1.57, 1.69
*P*- trend^c^			<0.001				0.890	
*P*- trend^d^			<0.001				0.462	
**Cholesterol (mmol/L)**								
Nulliparous women	3,011	4.95	5.35	5.27, 5.42	2 009	6.73	6.56	6.50, 6.63
Parous women	11,666	5.27	5.19	5.13, 5.25	9 702	6.58	6.50	6.45, 6.55
*P-*value^b^			<0.001				0.06	
*Months of lifetime lactation*								
Never	360	5.56	5.47	5.35, 5.59	345	6.48	6.47	6.33, 6.61
1–6	2,587	5.38	5.37	5.29, 5.45	1,834	6.52	6.51	6.42, 6.59
7–12	3,038	5.28	5.31	5.23, 5.39	2,363	6.54	6.52	6.43, 6.60
13–23	3,513	5.20	5.23	5.16, 5.31	2,753	6.61	6.58	6.49, 6.66
≥ 24	2,168	5.17	5.20	5.12, 5.28	2,407	6.62	6.51	6.42, 6.60
*P*- trend^c^			<0.001				0.362	
*P*- trend^d^			<0.001				0.552	
**HDL-cholesterol (mmol/L)**								
Nulliparous women	3,011	1.47	1.48	1.46, 1.51	2 009	1.47	1.50	1.48, 1.52
Parous women	11,666	1.44	1.42	1.39, 1.44	9 702	1.46	1.45	1.43, 1.46
*P-*value^b^			<0.001				<0.001	
*Months of lifetime lactation*								
Never	360	1.39	1.38	1.34. 1.43	345	1.46	1.44	1.39. 1.48
1–6	2,587	1.41	1.41	1.39. 1.44	1,834	1.48	1.45	1.42. 1.48
7–12	3,038	1.44	1.43	1.40. 1.46	2,363	1.47	1.45	1.42. 1.48
13–23	3,513	1.45	1.44	1.41. 1.47	2,753	1.46	1.45	1.42. 1.48
≥ 24	2,168	1.45	1.43	1.40. 1.47	2,407	1.41	1.43	1.40. 1.46
*P*- trend^c^			0.008				0.362	
*P*- trend^d^			0.06				0.265	
**LDL-cholesterol**^**e**^**(mmol/L)**								
Nulliparous women	2,999	3.15	3.49	3.42, 3.56	1 943	4.76	4.58	4.52, 4.65
Parous women	11,576	3.48	3.43	3.37, 3.49	9 471	4.64	4.57	4.52, 4.62
*P-*value^b^			0.006				0.661	
*Months of lifetime lactation*								
Never	353	3.77	3.70	3.58, 3.83	337	4.55	4.55	4.42, 4.69
1–6	2,564	3.60	3.60	3.52, 3.68	1,798	4.57	4.57	4.49, 4.66
7–12	3,021	3.49	3.53	3.46, 3.61	2,316	4.58	4.58	4.50, 4.67
13–23	3,482	3.41	3.45	3.38, 3.53	2,677	4.64	4.64	4.55, 4.72
≥ 24	2,156	3.40	3.44	3.36, 3.52	2,343	4.58	4.58	4.49, 4.67
*P*- trend^c^			<0.001				0.396	
*P*- trend^d^			<0.001				0.509	
**Glucose (mmol/L)**								
Nulliparous women	3,011	4.93	5.10	5.04, 5.16	2,009	5.61	5.52	5.46, 5.59
Parous women	11,666	5.02	4.99	4.94, 5.04	9,702	5.53	5.57	5.52, 5.62
*P-*value^b^			<0.001				0.183	
*Months of lifetime lactation*								
Never	360	5.15	5.10	5.00, 5.20	345	5.51	5.62	5.48, 5.76
1–6	2,587	5.04	5.02	4.96, 5.09	1,834	5.43	5.53	5.44, 5.62
7–12	3,038	5.01	5.00	4.94,5.06	2,363	5.51	5.58	5.49, 5.66
13–23	3,513	5.02	5.00	4.94, 5.07	2,753	5.54	5.59	5.50, 5.67
≥ 24	2,168	5.01	4.99	4.92, 5.06	2,407	5.60	5.57	5.48, 5.66
*P*- trend^c^			0.06				0.587	
*P*- trend^d^			0.232				0.268	

Abbreviations: No. number; CI, confidence interval; HDL, high density lipoprotein; LDL, low density lipoprotein.

^a^ For nulliparous women: Adjusted for maternal age, smoking status, physical activity, education, marital status and time since last meal. For parous women: Adjusted for maternal age, smoking status, physical activity, education, marital status, time since last meal and parity.

^b^*P*-value between nulliparous vs parous. Adjusted for maternal age, smoking status, physical activity, education, marital status and time since last meal.

^c^*P*-trend across all five categories of lifetime lactation duration, including the category “never”.

^d^*P*-trend across four categories of lifetime lactation duration, excluding the category “never”.

^e^LDL was calculated only if serum triglycerides were lower than 4.5 mmol/L.

Contrary to blood pressure and serum lipids, there was no statistically significant linear trend across the lactation categories in blood glucose levels (Table 3) in either of the two age groups. However, in women 50 years or younger there was weak evidence of a dose-dependent association (*P*-trend, = 0.06), but no associations remained after additional adjustments for body mass index (data not shown).

Of the total study sample of parous women (n = 21,368), 39.0% of the women had hypertension, 18.7% were obese and 2.5% had known diabetes (Table 4). The corresponding prevalences among nulliparous women (n = 5,020), were 37.5%, 17.0% and 3.0%, respectively. Among women 50 years or younger, lifetime duration of lactation was inversely associated with the prevalence of hypertension (*P*-trend, < 0.001), obesity (*P*-trend, < 0.001) and diabetes (*P*-trend, = 0.004). Parous women 50 years or younger who had never lactated had an almost doubled risk for hypertension, more than three times the risk of obesity and more than five times the risk of diabetes compared to women in the reference group who had lactated for ≥ 24 months (Table 4). Among women older than 50 years no associations were found. Adjusting for time since last birth did not change these estimates, whereas adjusting for body mass index attenuated the estimates of both risk for hypertension and for diabetes (data not shown).

**Table 4:** Odds ratio for hypertension, obesity and diabetes in nulliparous and parous women (n = 26,388)

	**Women ≤ 50 years of age (n = 14,677)**					**Women > 50 years of age (n = 11,711)**				
			**Crude OR**	**Adjusted OR**^**a**^	**95 % CI**			**Crude OR**	**Adjusted OR**^**a**^	**95 % CI**
**Hypertension**^**b**^	No.	No. hypertension				No.	No. hypertension			
Nulliparous women	3,011	367	1.0 (Ref.)	1.0 (Ref.)		2,009	1,514	1.0 (Ref.)	1.0 (Ref.)	
Parous women	11,666	2,006	1.49	0.75	0.65, 0.89	9,702	6,331	0.61	0.88	0.77, 1.00
*P*-value				<0.001					0.05	
*Months of lifetime lactation*										
Never	360	98	2.08	1.88	1.41, 2.51	345	110	0.85	1.26	0.96, 1.65
1–6	2,587	489	1.29	1.24	1.03, 1.49	1,834	719	0.61	0.88	0.75, 1.02
7–12	3,038	528	1.17	1.16	0.98, 1.37	2,363	864	0.69	0.93	0.80, 1.07
13–23	3,513	560	1.05	1.03	0.88, 1.21	2,753	996	0.70	0.89	0.78, 1.01
≥ 24	2,168	331	1.0 (Ref.)	1.0 (Ref.)		2,407	682	1.0 (Ref.)	1.0 (Ref.)	
*P*- trend^c^				<0.001					0.944	
*P*- trend^d^				0.009					0.218	
**Obesity**^**e**^	No.	No. obesity				No.	No. obesity			
Nulliparous women	3,011	357	1.0 (Ref.)	1.0 (Ref.)		2,009	497	1.0 (Ref.)	1.0 (Ref.)	
Parous women	11,666	1,501	1.10	0.82	0.70, 0.96	9,702	2 490	1.05	1.26	1.11, 1.43
*P*-value				0.013					<0.001	
*Months of lifetime lactation*										
Never	360	100	3.29	3.37	2.51, 4.51	345	97	0.87	1.17	0.89, 1.53
1–6	2,587	398	1.56	1.68	1.36, 2.06	1,834	429	0.68	0.92	0.79, 1.09
7–12	3,038	420	1.37	1.46	1.21, 1.77	2,363	552	0.68	0.88	0.76, 1.01
13–23	3,513	356	0.96	1.02	0.85, 1.23	2,753	668	0.72	0.89	0.78, 1.01
≥ 24	2,168	227	1.0 (Ref.)	1.0 (Ref.)		2,407	744	1.0 (Ref.)	1.0 (Ref.)	
*P*- trend^c^				<0.001					0.861	
*P*- trend^d^				<0.001					0.457	
**Diabetes**^**f**^	No.	No. diabetes				No.	No. diabetes			
Nulliparous women	3 011	20	1.0 (Ref.)	1.0 (Ref.)		2 009	129	1.0 (Ref.)	1.0 (Ref.)	
Parous women	11 666	77	0.99	0.59	0.32, 1.11	9 702	466	0.74	1.01	0.80, 1.26
*P*-value				0.102					0.957	
*Months of lifetime lactation*										
Never	360	10	5.60	5.87	2.25, 15.3	345	21	0.71	1.29	0.78, 2.14
1–6	2 587	18	1.37	1.49	0.63, 3.53	1 834	64	0.57	0.71	0.50, 1.01
7–12	3 038	19	1.23	1.29	0.57, 2.89	2 363	91	0.51	0.74	0.55, 1.00
13–23	3 513	19	1.07	1.06	0.48, 2.33	2 753	131	0.92	0.89	0.69, 1.16
≥ 24	2 168	11	1.0 (Ref.)	1.0 (Ref.)		2 407	159	1.0 (Ref.)	1.0 (Ref.)	
*P*- trend^c^				0.004					0.202	
*P*- trend^d^				0.292					0.014	

Abbreviations: No, number; OR, odds ratio; CI, confidence interval, Ref., reference category.

^a^ Adjusted for maternal age, smoking status, physical activity, education and marital status. For parous women: maternal age, smoking status, physical activity, education, marital status and parity.

^b^Hypertension defined as ≥ 140/90 mmHg or current antihypertensive treatment.

^c^*P*-trend across all five categories of lifetime lactation duration, including the category “never”.

^d^*P*-trend across four categories of lifetime lactation duration, excluding the category “never”.

^e^Obesity defined as BMI ≥ 30 kg/m.

^f^Diabetes defined as blood glucose ≥ 11.1 mmol/L or self reported diabetes in the questionnaire.

## Discussion

In this large population-based study, we found that prolonged lifetime lactation was associated with a more favourable cardiovascular risk profile among women 50 years or younger. Parous women ≤ 50 years who had never lactated were more likely to have developed hypertension, obesity and diabetes than women who had the longest lactation duration. Furthermore, there were strong indications of a dose–response association between the total duration of lactation and a favourable cardiovascular risk profile. Although the largest difference was found for women who had never lactated compared to those who had ever lactated, our analyses showed that the associations remained significant also within the lactation categories. Among women older than 50 years, only waist circumference and possibly diabetes were associated with lactation duration.

Our findings are consistent with recent studies showing that the favourable effects of lactation on maternal metabolic health persist post weaning [7,8,11,17-20] and thereby further supporting the notion that lactation may induce long-term beneficial effects on maternal blood pressure, weight [21], diabetes [6,18], components of the metabolic syndrome [5,19] and cardiovascular health [7,11,17]. Previous studies have shown that the beneficial effect of lactation on cardiovascular risk factors seems to wane with time since last birth [6,11,18]. Adjusting for this period did not change the estimate in our study. On the other hand, the stronger associations observed among women aged ≤ 50 years compared to those aged >50 years could possibly be due to the shorter period since last birth.

The present study was conducted in a large and unselected population with a wide age range and a high participation rate. Breastfeeding was common, and this observation is consistent with other studies showing that breastfeeding rates in Norway are among the highest in industrialised countries [22]. We therefore have a large sample size of women who have lactated. Combined with the standardised measurements of lipids, anthropometric measures and blood pressure, it provides a unique opportunity to study the association of lactation and cardiovascular risk factors and whether differences exist by duration of lactation.

However, the cross sectional study design calls for a cautious interpretation of the findings, as in all observational studies. It is possible that women who breastfeed their children have a better health status, healthier lifestyles and higher socioeconomic status than women who do not breastfeed [23]. Given the high rates of breastfeeding among Norwegian women, the group of women who had never lactated in our study was less than 4% of the entire sample. Thus, it is possible that the group of women who had never lactated in our study differed in major confounders than might be expected in populations where breastfeeding rates are lower.

A previous study among Norwegian women found that maternal age, education and smoking were among the most important factors associated with lactation duration [22]. In addition to these factors, we found significant differences across the lactation categories in the level of physical activity, marital status, and parity in our study. Although all of these factors are known to be associated with risk of cardiovascular disease, adjusting for them did not materially change the estimated associations. However, residual confounding due to unmeasured and unknown factors cannot be ruled out, such as pre-pregnancy and early postpartum health status. Women with gestational diabetes mellitus are at an increased risk of developing type 2 diabetes [24]. Furthermore, gestational diabetes mellitus may have a role in impacting breastfeeding initiation and success, and could thus act as a major confounder. In a recent study, longer duration of lactation was associated with lower incidence of the metabolic syndrome both among women with and without a history of gestational diabetes mellitus, and the findings were particularly striking for women who developed gestational diabetes mellitus during their pregnancy [10]. In our study, women reporting a diagnosis of diabetes prior to first pregnancy were excluded from our analyses. Nevertheless, the lack of data on the history of gestational diabetes mellitus during pregnancy is a limitation of our study.

Moreover, the potential for reverse causation must be considered when interpreting the results from the present study*.* Obesity [25,26] and type 1 diabetes [27] have been linked to difficulties with lactation, and hence shorter lactation duration could be a marker for an already existing abnormal metabolic profile influencing whether the women lactate and for how long. Unfortunately, we did not have pre-pregnancy measurements of weight and height and could therefore not adjust for pre-pregnant body mass index. However, when we adjusted for body mass index measured at study participation in supplementary analyses, the adjustments did not change our estimated associations substantially, with the exception of HDL-cholesterol. Obesity may either precede [26] or follow lactation practices. Thus, one may argue that body mass index measured at study participation rather acts as an intermediate factor, and hence should not be adjusted for as a confounder in the analyses.

Diet accounts for much of the variation in coronary heart disease risk [28]. The HUNT2 study was not designed to measure dietary intake, and we had insufficient dietary data to adjust for dietary factors in our analyses. However, previous studies have found that the association between lactation and cardiovascular health persists even after adjustment for dietary intake [5,7,11,17].

Another limitation of the study is the lack of data on lactation intensity. Higher intensity of lactation has been associated with improved fasting glucose and lower insulin levels at 6–9 weeks postpartum in a previous study [29]. Data on lactation intensity could therefore possibly have strengthened our estimates of associations among women with higher, and attenuated the associations among women with lower, lactation intensity. Moreover, lactation was assessed retrospectively. Nevertheless, studies have shown that maternal recall of lactation is fairly valid and reliable [30], even after 20 years [31]. However, even if misclassification should exist, it is not likely to be differential according to cardiovascular risk factors. Our observed estimates are therefore likely to be conservative.

Furthermore, selection bias could have influenced our results. However, a non-responder study showed that the most important reason for non-attending the HUNT2-study in the age group 20–69 was lack of time/moved away, while in those aged 70 years or more, immobilising and frequent follow-up by medical doctor were important reasons [32]. We do not believe that reasons for non-attending were unevenly distributed across the lactation categories, and we find it unlikely that selection bias would have altered the results in our study.

During pregnancy the maternal metabolism is profoundly changed, and the changes that occur could theoretically increase women’s risk of metabolic disease. These changes include accumulation of adipose tissue stores [33], increased insulin resistance [34] and blood pressure, [35] as well as a change of the quantity and quality of circulating lipoproteins [36,37]. By the end of the pregnancy, LDL cholesterol and triglyceride levels are two to three times higher compared with pre-pregnancy levels. In fact, some studies have shown that increasing parity may increase risk of cardiovascular disease [38,39]. These studies do not, however, include data on lactation. Our findings of a more favourable cardiovascular risk profile associated with lactation seem to confirm the recent suggestion that lactation could affect risk of metabolic disease by facilitating a faster resetting of the maternal metabolism after pregnancy [40].

Lactation increases a mother’s metabolic expenditure by an estimated 480 kcal/d [41], and although the association between lactation and postpartum weight loss so far remains inconclusive [21,42-45], lactation could reduce cardiovascular risk by mobilising accumulated fat stores. Furthermore, lactation provides a route for physiologic excretion of large amounts of cholesterol, which could explain the more speedy return of blood lipids to pre-pregnancy levels observed in lactating mothers [3]. Additionally, hormonal effects, such as those of prolactin and oxytocin, may affect maternal blood pressure [46]. Our data among women with an average time since last pregnancy of about 21 years suggest that these favourable changes are persisting on a long term scale and are not limited to the period of lactation. Among women older than 50 years, however, we found no similar linear trend in the association between lifetime duration of lactation and cardiovascular risk factors as in younger women. Still, women > 50 years who had never lactated had a significantly higher body mass index, wider waist circumference, higher lipid and glucose levels and higher prevalence of hypertension, obesity and diabetes compared to women who had lactated. Menopause appears to be a time of transition to increased cardiovascular risk, including adverse changes in serum lipid profile [47]. Hence, the cardiovascular risk alterations occurring during the menopausal transition may dilute the possible beneficial effects of lactation on maternal metabolic health as shown in previous studies [6,11,18].

Lactation may also improve insulin sensitivity and glucose tolerance. Insulin levels and insulin/glucose ratios are lower, and carbohydrate use and total energy expenditure are higher, in the lactating women compared to women who do not lactate [41]. Our data suggest a relation between lactation and glucose levels later in life. However, no statistically significant association with life-time duration of lactation could be found in either age group, although the association among women 50 years or younger were close to significant. In contrast, the association between lifetime duration of lactation and the prevalence of diabetes was strong and significant in the younger age group, although not among the older women, further supporting the notion that the possible effect wanes with time since last delivery. These mechanisms, together with our results, indicate that lactation helps women return to pre-pregnant metabolism more quickly post partum, which could in turn affect metabolic disease risk profile later in life.

Our results indicate that lactation may have a considerable impact on cardiovascular risk factors. The difference in systolic/diastolic blood pressure between women 50 years or younger who had never lactated and women who had lactated for 24 months or more is similar to the blood pressure-lowering effect of salt reduction (4/2 mm Hg) among normotensive individuals [48]. Furthermore, it has been estimated that a 10% reduction in serum cholesterol could halve the risk of ischaemic heart disease at age 40 [49], and hence the *5* % difference in total cholesterol levels observed between women 50 years or younger who had never lactated, and women who had lactated more than 24 months, could represent a substantial risk reduction. Also, the 17% difference in triglycerides between women 50 years or younger who had never lactated and women who had lactated 24 months or more must be added to this altered cardiovascular disease risk pattern.

## Conclusions

In conclusion, this large population-based study showed that lactation is associated with a more favourable cardiovascular risk profile in mothers later in life, and that the beneficial effects are most prominent among women 50 years or younger. Lactation may hence reduce the adverse pregnancy-related changes in cardiovascular risk factors, with effects lasting even beyond the childbearing years. If the observed associations are causal, lactation could have substantial potential for reducing women’s risk of cardiovascular disease. Additional studies are needed to confirm the observed protective associations and their underlying mechanisms.

## Competing interests

The authors declare that they have no competing interests.

## Authors’ contributions

STN conceived the idea, did the analyses and wrote the paper. KM participated in the planning of and data collection in the HUNT2 Study. SF, TILN, LFA and KM participated in the analyses, interpreted the results and wrote the paper. All authors discussed and interpreted the findings and contributed to the final paper.
